# The Pineal Body

**Published:** 1913-02-15

**Authors:** 


### THE PINEAL BODY.

At a particular place on the upper surface of the primitive tubular brain which characterises all vertebrates there is an excrescence known as the pineal body or pineal gland. In mammals this outgrowth is comparatively insignificant in size, and is completely hidden from view, when the skull is opened, by the enormous development of the cerebral hemispheres and other parts of the brain. Nevertheless, the pineal body exists in all vertebrate animals except a very few degenerate species at the lowest end of the scale; and it can be traced through fossils to a very remote antiquity. The Darwinian theories and the new light which they shed on embryology and phylogeny invested the pineal body with remarkable interest; for it was then recognised that the organ is, in fact, the vestigial relic of a median eye. Classically minded biologists did not hesitate to recall the story of the Cyclops, and to suggest that that legendary Homeric character was perhaps drawn from a vague tradition of some creature which actually used a "pineal eye" situated in the middle of its forehead.

There, for a space, the pineal body was left stranded, labelled as a useless relic of a long-forgotten usefulness. But lately investigators have turned their attention to the pineal body once more, struck by the remarkable fact that this despised rudiment has managed to hold its place from palæozoic times down to the present day. If it were really as functionless as was once supposed, surely it would have disappeared long ago, it was argued. And so it has turned out; for careful research has proved that the pineal body has very important functions indeed, though the full detail of all that it does has not yet been completely marked out. Most of the work has been done abroad; and hitherto the literature on the subject in English has been very meagre. In the *Medical Chronicle* Dr. Leonard Kidd removes this reproach, and reviews in great detail all that recent years have disclosed concerning the mysterious pineal gland. Pineal medication, as he says, is still in the clouds, and is probably the only form of organotherapy which has never been tried. If it ever comes (surely it will, Dr. Kidd), he hopes it will follow, not precede, the experimental study for which he pleads.

What, then, are the known facts at present about the pineal body and its properties? It is fairly safe to agree with Dr. Kidd that it is a metamorphosed, not a rudimentary, useless, degenerating, or disappearing organ; and that it probably furnishes an internal secretion, analogous to those of the thyroid, pituitary, etc. His further conclusions are also justified by such facts as have been ascertained so far, though the next few years may possibly modify some of them. Thus he holds that the pineal body of very young birds and mammals has an inhibitory action on the development of the testes, and through them on bodily growth and the appearance of the secondary sexual characters. The relation of the pineal body to the ovaries is less clear, and further research is required before a definite statement can be made. To the pituitary and the adrenal relationship is probable, to the thyroid and thymus possible; but on these points nothing certain has been established. It also seems likely that the pineal has some other function besides the prepubertal, and that this other function goes on right up to old age; for a partial involution occurs at puberty, corresponding to the waning of the one function, while other elements of the gland remain fully developed throughout life. Indeed, Dr. Kidd thinks it possible that the pineal has three functions, not two; but he admits that at present it is not possible to say how the gland functionates. At any rate, it is clear that a splendid field is open for research, and the voluntary hospitals offer the best opportunities of clinical material to those who set themselves to find the clue to the problems involved. It is interesting also to note that almost the only British case of pineal tumour which Dr. Kidd quotes comes from one of the London general hospitals.

